# A novel analytical approach to quantitative myocardial edema imaging in acute myocarditis using T2-mapping

**DOI:** 10.1186/1532-429X-18-S1-W9

**Published:** 2016-01-27

**Authors:** Bettina Baessler, Frank Schaarschmidt, Bernhard Schnackenburg, Christian Stehning, Melanie Treutlein, Anastasia Dick, Guido Michels, David Maintz, Alexander Bunck

**Affiliations:** 1 grid.411097.a000000008852305XDepartment of Radiology, University Hospital of Cologne, Cologne, Germany; 2 grid.9122.80000000121632777Institute of Biostatistics, Faculty of Natural Sciences, Leibniz Universität Hannover, Hannover, Germany; 3 Philips Healthcare Germany, Hamburg, Germany; 4 grid.418621.80000000403734886Philips Research Europe, Hamburg, Germany; 5 grid.411097.a000000008852305XDepartment III of Internal Medicine, Heart Centre, University Hospital of Cologne, Cologne, Germany

## Background

The purpose of this study was to investigate the diagnostic value of T2-mapping in patients with acute myocarditis (ACM) and to define an appropriate cut-off value for quantitative edema detection.

## Methods

CMR data of 35 patients with clinically suspected ACM and confirmation of diagnosis by CMR according to the Lake Louise criteria (LL criteria) were retrospectively analyzed. 30 healthy volunteers (HV) served as a control. A second cohort consisting of 72 patients with clinically diagnosed ACM served as a validation cohort. All patients and HV were examined on a clinical 1.5T scanner, where - in addition to the routine CMR protocol - a Gradient Spin Echo (GraSE) T2-mapping sequence had been acquired at two basal, midventricular and apical slices in short axis view. T2-maps were segmented according to the 16-segments AHA-model. While averaging all pixels within one myocardial segment for segmental T2-calculation, their standard deviation ("pixel-SD") within segments was recorded as an indicator for the "spottiness" of T2 values, originating from the spatial variation of myocardial edema in ACM. Statistical analysis was conducted using single and multiple logistic regression analyses, random forests, decision trees, and ROC-analyses.

## Results

**Figure 1:**
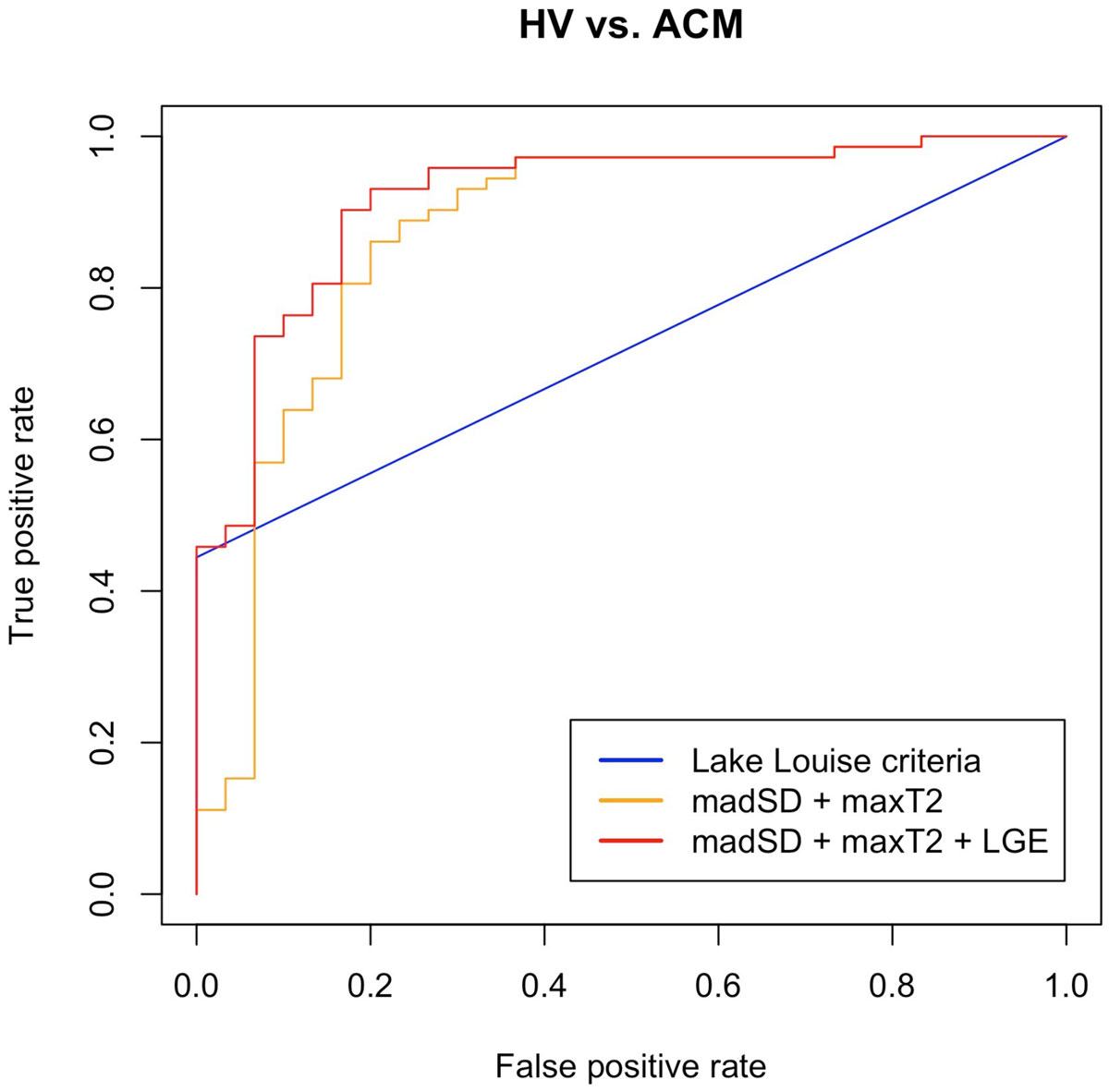
**ROC-analysis for differentiating healthy volunteers from ACM patients**. LL Criteria (recorded as "present"/"not present"): Late Gadolinium Enhancement (LGE) + Visual edema on T2 black-blood images + Early Gadolinium Enhancement

Means of global myocardial T2 or pixel-SD showed large overlaps between HV and patients with CMR-proven ACM. Variation of T2 values as well as of pixel-SD, however, was much larger in ACM patients compared to HV. In random forests and multiple logistic regression analyses, the combination of the highest segmental T2 value within each patient (maxT2) and the mean absolute deviation of pixel-SD (madSD) over all 16 segments within each patient proved to be the best discriminators between HV and patients with CMR-proven ACM. In decision trees, a cut-off of 2.5 ms for madSD and of 67.7 ms for maxT2 resulted in 90% sensitivity and 67% specificity for classification between HV and CMR-proven ACM when one of the two criteria was met. Applying these cut-offs on the validation cohort (n = 72) resulted in equal diagnostic sensitivity and specificity. A re-estimation of cut-offs in the validation cohort led to slightly different cut-offs of 1.7 ms for madSD and of 68.9 ms for maxT2 with a sensitivity of 81% (97%) and a specificity of 80% (63%) for classification between HV and ACM when both criteria (one of the two criteria) were met. In ROC-analyses, the combination of madSD and maxT2 showed superior diagnostic performance (AUC 0.87) when compared to LL criteria (AUC 0.72; Figure 1), and the combination of madSD, maxT2 and Late Gadolinium Enhancement (LGE) even resulted in an AUC of 0.92.

## Conclusions

The proposed cut-off values for maxT2 and madSD in the setting of ACM allow edema detection with high sensitivity and specificity and in a quantitative manner. The two parameters show an additional diagnostic value to LGE with the inherent potential to overcome the hurdles of T2-mapping for its integration into clinical routine.

